# Statistical estimation of noise induced hearing loss among the drivers in one of the most polluted cities of India

**DOI:** 10.1038/s41598-024-55906-9

**Published:** 2024-03-25

**Authors:** Manish Kumar Manar, Shivendra Kumar Singh, Prashant Kumar Bajpai, Veerendra Verma, Sheo Prasad Shukla, Neeraj Kumar Singh, Markandeya

**Affiliations:** 1 Department of Community Medicine and Public Health, King George’s Medical University, Lucknow, 226003 India; 2 Department of Otorhinolaryngology, King George’s Medical University, Lucknow, 226003 India; 3 Rajkiya Engineering College, Banda, 210201 India; 4 Central Mine Planning and Design Institute Limited (CMPDIL), Regional Institute-7, Bhubaneswar, 751013 India; 5 Ex-Department of Civil Engineering, Indian Institute of Technology (BHU), Varanasi, 221005 India

**Keywords:** Noise pollution, Noise induced hearing loss, Noise dose, Cumulative noise exposure, Dose–response relationship, Environmental sciences, Environmental social sciences, Engineering

## Abstract

In the present study, an attempt has been made to assess the impact of vehicular noise upon the 3-wheeler tempo drivers and to know whether there is any relationship between hearing loss and cumulative noise exposure. For this purpose, 3-wheeler tempo drivers (Exposed group) and non-commercial light motor vehicle car drivers (Unexposed group) were chosen as study subjects. Three traffic routes were selected to assess the noise level during waiting and running time in the exposed and unexposed groups. Among all three routes, the highest mean noise level (L_eq_) was observed on the Chowk to Dubagga route for waiting and en-route noise measurement. It was measured as 84.13 dB(A) and 86.36 dB(A) for waiting and en-route periods of 7.68 ± 3.46 and 31.05 ± 6.6 min, respectively. Cumulative noise exposure was found to be significantly different (*p* < 0.001) in all age groups of exposed and unexposed drivers. Audiometric tests have been performed over both exposed and unexposed groups. The regression analysis has been done keeping hearing loss among tempo drivers as the dependent variable and age (years) and Energy (Pa^2^ Hrs) as the independent variable using three different criteria of hearing loss definitions, i.e., World Health Organization, National Institute for Occupational Safety and Health (NIOSH), Occupational Safety and Health Administration criteria. Among these three criteria, the NIOSH criterion of hearing loss best explained the independent variables. It could explain the total variation in dependent variable by independent variable quite well, i.e., 68.1%. The finding showed a linear relationship between cumulative noise exposures (Pa^2^ Hrs) and the exposed group's hearing loss (dB), i.e., hearing loss increases with increasing noise dose. Based on the findings, two model equations were developed to identify the safe and unsafe noise levels with exposure time.

## Introduction

Noise is one of the most commonly present environmental pollutants in the workplace as well as in communities, affecting the health of millions of people around the world^1^. It is defined as undesirable, unwanted, or unpleasant sound, which is produced due to pressure changes in the medium (usually air) caused by turbulence or vibrations^2,3^. The human ear is most sensitive to the sound frequencies between 500 and 4000 Hz, less sensitive at higher and much less sensitive at low. This sound range sensitivity of the human ear coincides with the range of voice communication^4^. Pollution due to noise is considered one of the primary forms of urban pollution prevalent in major cities worldwide^5,6^.

Urban noise pollution is a primary environmental concern worldwide and its significance is increasing as the world becomes more urbanized. Many studies have been conducted worldwide to assess and document the ill effects of noise exposure^3,7,8^. Some comprehensive reviews on noise exposure and its health effects documented various health effects, e.g., Hearing loss, annoyance, poor sleep, elevated blood pressure, mental disorders, etc.^9^. Nandi and Dhatrak^10^ studied on occupational noise-induced hearing loss in India and concluded that exposure to excessive noise is major cause of permanent hearing loss. Xie et al.^11^ studied the effect of cumulative noise exposure (CNE) on high frequency hearing loss of the employees of two plants of Zhejiang, China. Otological examination followed by pure tone audiometry to assess hearing threshold shift at different frequencies 3000, 4000 and 6000 Hz was done to test the hearing status of the subjects. Carter et al.^12^ studied the lifetime noise exposure due to night clubbing, rock concerts and music festivals on adolescents and young adult Australians. Zhang et al.^13^ conducted a cross-sectional study in Shiyan city, China in two large automobile manufacturer industries. Total 205 workers were included in that study. Personal noise dosimeter was used to assess the noise exposure; occupational noise history was recorded through interview as well as records of the employer. Audiometry of the employee was done after a quit period of 16 h. They also reported that 88.3% of employees were exposed to more than 85 dB(A) of noise exposure. The lifetime CNE estimated f or each employee and found in the range of reported CNE ranging from 86.4 to 112.5 dB(A) years.

Sharif et al.^14^ studied the hearing status of traffic police personnel with or without sound protector. This was a cross-sectional study conducted in Dhaka. Ansari et al.^15^ evaluated the hearing loss among drivers in Zahedan, Iran. The study was performed among 1836 drivers in the year 2013. Hearing loss in both ears was measured at 250, 1000, 2000, 3000, 4000, 6000 and 8000 Hz. Beheshti et al.^16^ determined the prevalence of hearing loss among taxi and agency drivers.

On the other hand, road traffic is also one of the significant and most pervasive sources of urban community noise, particularly in the modern urban environments of many nations, where dense traffic systems are very well-developed^10,17^. Road traffic is known to play a dominant role in the current noise scenario and affects more people than other forms of transportation. It is estimated that road traffic noise accounts for about 78% of the total noise in the urban environment^18,19^. In the present study, Lucknow, the capital city of Uttar Pradesh was taken into consideration. Due to rapid urbanization in last few decades this city observes a sudden growth in vehicular traffic which ultimately responsible for urban noise pollution. Those who spend most of their time near the road, such as roadside residents, shopkeepers, traffic police personnel, drivers, etc., are most affected by traffic noise^20,21^. Drivers of vehicles are supposed to be the most noise-exposed population of traffic noise as they spend most of their time virtually on the noise source (own vehicle noise) and remain in the vicinity of the traffic flow^22^. In various forms, noise can have a range of ill effects on individuals, communities and the environment, such as hearing loss, tinnitus, stress and anxiety, sleep disturbances, reduced awareness and concentration, increased blood pressure etc.^23^.

Different organizations like Environmental Protection Agency (EPA), WHO, NIOSH and OSHA have given noise exposure limit at the work place considering uniform noise exposure duration of 8 h. But in real life situations the exposure levels and durations are not same and logarithmic nature of decibel unit of noise exposure make it difficult to mathematically calculate safe exposure duration. In view of the above facts, an attempt has been made to understand the relationship between cumulative noise exposure and noise induced hearing loss.

## Material and methods

The study was approved by the Institutional Ethics Committee of King George’s Medical University Lucknow, vide letter No. 2061/Ethics/R.cell-17 Dated 20/11/17. The registration no. of the ethics committee is ECR/262/Inst/UP/2013/RR-16 and reference code for the said approval is 86th ECM II B-Ph.D./P3. All methods were performed in accordance with the relevant guidelines and regulations. Informed consent was obtained from all subjects and their legal guardian(s).

### Study area

**Figure 1:**
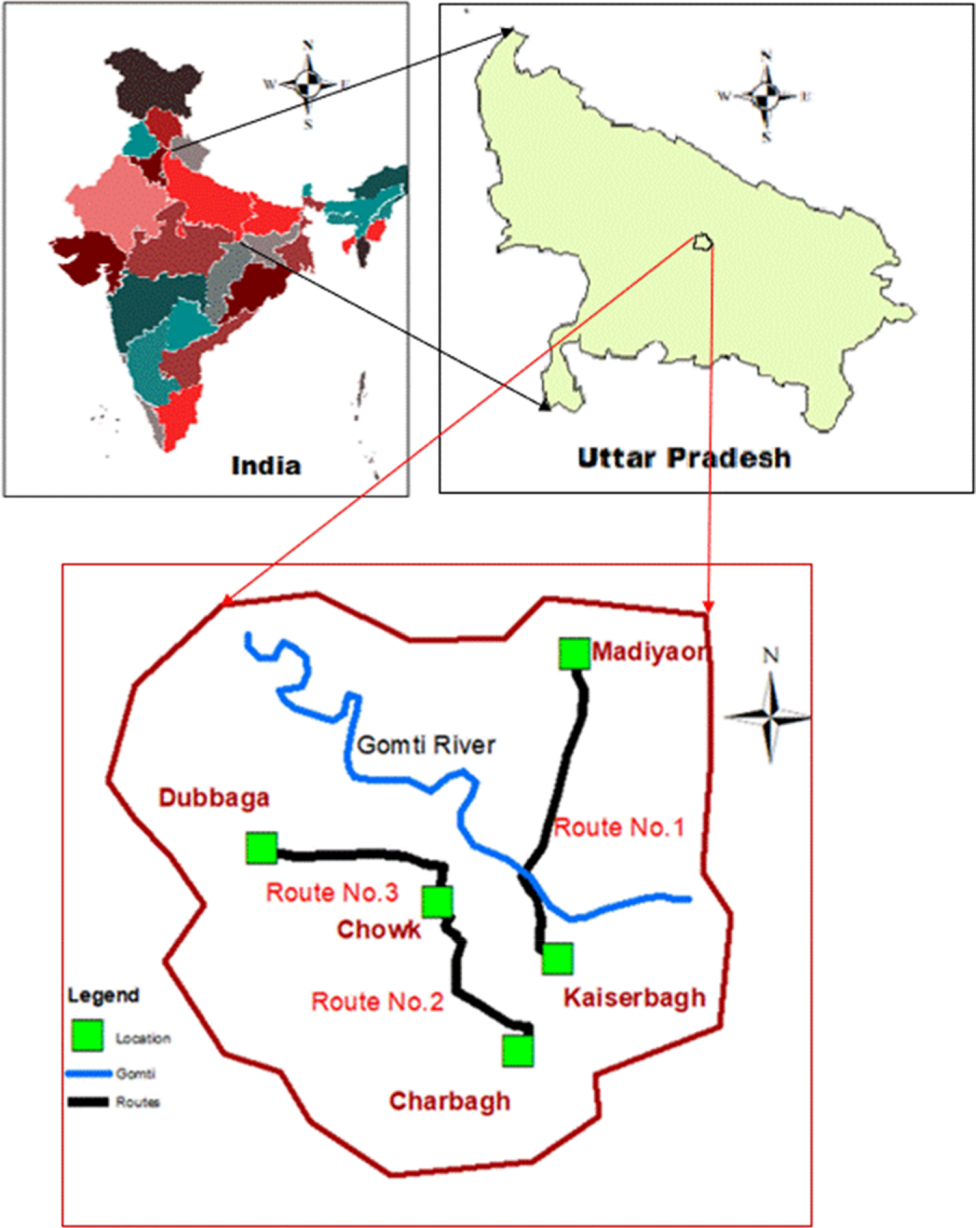
Study route for noise sampling (Digitized using ArcGIS version 9.2).

Lucknow city has expanded its boundary exponentially in the last two decades resulted in traffic load, increase in population and associated services but in the old city, the population, nature of the traffic and type of public transport has not changed significantly. A total number of 3-routes have been selected from old Lucknow that were (1) Kaiserbagh- Madiyaon (Distance 8 km, number of tempo and fuel type: 15 CNG and 15 Diesel), (2) Chowk- Charbagh (Distance 6 km, number of tempo and fuel type: 15 CNG), (3) Chowk- Dubagga (Distance 7.5 km, number of tempo and fuel type: 15 Diesel) as shown in Fig. 1. In selecting the above routes, the following factors of the roads which may influence the traffic noise as well as noise inside the vehicles were also considered: (1) to get the drivers having maximum driving experience, (2) no new type of public transport inducted, (3) no major road repair or constructions were in progress, (4) the routes are not significantly widened or nature of road (bitumen paved, concrete or tiled etc.) changed in the past.

The information of the routes was collected through detailed enquiry about road traffic diversions due to construction activities or other reasons (e.g., road traffic hindrance from recently constructed bicycle track) were also done through travelling on different routes and enquiry from public, drivers, roadside shopkeepers, and traffic police personnel.

### Sampling size of vehicles for noise estimation

For noise sampling, three types of vehicles i.e., Diesel three-wheelers, CNG 3-wheelers and light motor vehicles (LMV), were selected in this study. In a study conducted on Delhi traffic noise by Anyogita et al.^24^, 30 vehicles of each mode of transport were taken into consideration for noise measurement. Hence for this study also 30 vehicles each of diesel tempo (30), CNG tempo (30) and cars (30) were included for noise measurements.

### Sampling size of drivers for health assessment

The sample size of drivers for health assessment was calculated using the following formula as provided in Eq. (1). The prevalence of hearing loss was taken 22.36–22.00% among professional drivers. This was taken from the studied done by Lopes et al.^25^. The confidence interval was taken 95% (5% significance level) and the precision was taken as 7% after due deliberation with the experts to keep the sample size reasonable to perform the study.

1 $$N = \frac{4pq}{{d^{2} }}$$

where N = required sample size, p = prevalence, q = 1 − p, d = precision, Taking 5% significance level with 0.07 precision

2 $$N = \frac{{4 \times 0.22 \times 0.78{ }}}{0.07 \times 0.07} = 141.72$$

Thus, 150 drivers in each group i.e., 150 tempo and 150 car drivers were included in the study.

### Sampling technique for selecting drivers for health assessment

In this study multistage random sampling was used for the selection of tempo drivers for their health assessment and collection of relevant information. In the first stage, the major routes of old Lucknow for public transport of three wheelers were identified as discussed in Section “Study area”.

At second stage, a list of tempo drivers was obtained for these three routes from the respective contractors of the tempo stands. This list has basic information like age of driver, type of fuel used and contact number. A list of 250, 378 and 452 tempo drivers were sorted out for route 1, 2 and 3 respectively. After maximum effort, 231 out of 250, 319 out of 378 and 421 out of 452 tempo drivers for route 1, 2 and 3 could be traced respectively. Each of these tempo drivers were contacted and were explained about the purpose of the study. Out of these tempo drivers 221, 301 and 411 drivers gave consent for the study for route 1, 2 and 3 respectively. For route 1, these 221 drivers were again contacted and explained about the purpose of the study. Only 211 out of 221 tempo drivers gave consent to participate in the study. The same procedure was adopted for route 2 and route 3.

So finally for route (1) 211 Drivers, for route (2) 248 drivers and for route (3) 351 drivers gave consent for the study. Total Sample size calculated for tempo drivers was 150. So, we divided this in 45, 50 and 55 according to tempo driver’s population proportion for route (1), (2) and (3) respectively.

At third stage, for route (1), from the list of 211 driver every third subject was selected by systematic random sampling technique and this process was continued till 45 subjects were selected, after applying exclusion and inclusion criteria. In this context we have examined 57 subjects for route 1.

Same technique was used for Route (2) and (3). So, for selection of 50 subject 58 subjects and for selection of 55 subjects 63 subjects were examined for route (2) and (3) respectively.

Similarly, for unexposed drivers, a total number of 150 subjects have been identified. Personal cars of academicians and scientists from different academic and research institutions were selected by convenient sampling for assessment of noise exposure in the comparison group (unexposed group). A list of car drivers was contacted through the academicians and scientists and enrolled for the study who met the inclusion and exclusion criteria.

#### Inclusion and exclusion criteria

**For tempo drivers (exposed group)**

- Inclusion criteria.
  Tempo drivers who gave consent to participate in the study.
  Working as tempo drivers for at-least one year.
- Exclusion criteria
  Drivers aged less than 18 years and more than 50 years.
  Drivers who were previously exposed to non-traffic occupational noise.
  Drivers who have history of diabetes mellitus.
  Drivers with perforated tympanic membrane.
  Drivers who were prescribed ototoxic drugs.

**Inclusion and exclusion criteria for car drivers (unexposed group)**

- Inclusion criteria.
  Car drivers who gave consent to participate in the study.
  Working as a driver for at-least one year.
- Exclusion criteria.
  All exclusion criteria were same as tempo drivers. One additional criterion applied to exclude the drivers who ever worked as commercial vehicle driver.

### Noise sampling technique

A precision type-1 (IEC 651; BS 5969) sound level meter instrument (Quest Model 1900, U.S.A) was used for sampling of noise exposure among tempo drivers and non-commercial light motor vehicle (Car) drivers. At each route, noise measurement was made for two activities of the drivers:

a. Vehicle noise exposures among drivers while plying from one tempo stand to another.

b. Waiting area (tempo stand) noise exposures while waiting for passengers.

Thus, 30 CNG and 30 diesel tempos were selected for noise exposure assessment for the exposed group^23^. Tempos were chosen in all three routes in such a way that at least one tempo is selected at every hour interval for noise measurement from 7.00 AM to 10.00 PM to include noise variation of whole day.

The instrument was set to measure A-weighted (measures noise at frequencies to which the human ear is sensitive) sound levels in fast mode, i.e., one noise level reading in every second elapsed. The rear seat was occupied to sample noise in moving vehicles. Drivers were requested not to play music system during the running period. The instrument sensor was kept just behind the neck of driver and noise level measurements were recorded for the total duration elapsed while plying one tempo stand to another tempo stand. By reaching the destination tempo stand, recorded data was quickly saved in instrument memory and instrument was reset to record waiting area noise. The researcher followed the driver as closely as possible, keeping the instrument on “ON mode” to record of noise exposure of the driver while waiting for passengers. The noise recording was done until the tempo driver was again ready to ply the vehicle. By this procedure, one single trip of noise measurement is completed. The same procedure was followed for noise recording of all thirty trips of each type of tempos, i.e., thirty samples for diesel-driven tempos and thirty samples for CNG-driven tempos.

For non-commercial cars, windows of cars were kept closed; drivers were requested not to play music system. Care was taken to include various models and manufacturers of the cars. Before the selection of cars, number of noise measurements were done in different models, manufacturers and fuel types (Diesel/Petrol) of cars. It was practically not feasible to monitor noise exposure as per actual routes and routines of the car drivers. Therefore, a total of 15 min noise measurement inside vehicles of different models and at different routes of Lucknow city was done. Same kind of approach for in-vehicle noise assessment was used by other researchers^24,26^.

### Calculation of noise parameters

#### Time-weighted average (TWA) equivalent noise level (L_eq_)

It was observed that the in-vehicle equivalent noise level of diesel tempos of different routes was not significantly different; therefore time-weighted average equivalent sound pressure level (*L*_*Aeq*_) for diesel tempos was estimated using by calculating L_eq_ from following formula:

3 $$L_{eq} = 10\log \frac{1}{N}\mathop \sum \limits_{n = 1}^{n = N} \left( {10} \right)^{{L_{n} /10}}$$

where *L*_*eq*_ = Equivalent sound pressure level of an activity, in dB(A), *L*_n_ = *n*th sound pressure level of activity readings, in dB(A), n = 1, 2, 3, 4 … N.

Time-weighted average (TWA) equivalent noise level (*L*_*Aeq*_) was calculated from following formula:

4 $$L_{Aeq} = 10\log \frac{1}{T}\mathop \sum \limits_{i = 1}^{i = k} \left( {10} \right)^{{L_{eq} /10}} \times T_{i}$$

where T_i_ = ith duration of noise exposure, in seconds, T = ∑T_i_, in seconds, i = 1, 2, 3, 4 … k, k = Number of exposure activities.

#### Noise exposure energy

Noise exposure energy (Noise without decibel) is calculated by converting decibel into pascals squared seconds (Pa^2^.S), using a formula given by the American National Standard Institution^27^:

Noise Exposure energy ${(E}_{i})={p}_{{\text{exp}}(i)}^{2}\times {\mathrm{Length of exposure in seconds }({\text{t}}}_{{\text{i}}})$

5 $$P_{{{\text{exp}}\left( i \right)}}^{2} = 10^{{\left( {\frac{{L_{Aeq\left( i \right)} }}{10}} \right)}} \times P_{{{\text{ref}}}}^{2}$$

where ${P}_{{\text{exp}}}^{2}$ = A weighted pressure squared, in Pascal Squared, ${P}_{ref}^{2}$ = Reference pressure squared (P_ref_ = 20 μPa squared), in Pascal Squared, L_Aeq_ = A-weighted equivalent sound pressure level, in dB(A), *i* = Represents specific activity (e.g., En-route vehicles noise, waiting area noise).

#### Daily noise exposure energy

The daily noise exposures among tempo drivers and car drivers were estimated using Eq. (6) as given below:

6 $${\text{Total}}\;{\text{daily}}\;{\text{noise}}\;{\text{exposure}}\left( E \right) = \sum P_{{{\text{exp}}\left( i \right)}}^{2} \times T_{d} \left( i \right)$$

where ${P}_{{\text{exp}}(i)}^{2}$ = Sound pressure squared for the ith activity, in Pascals, T_d_(i) = Total duration spent in a day for ith activity, in seconds.

#### Cumulative noise exposure (CNE)

The cumulative noise exposure (CNE) is calculated by multiplying daily noise exposure energy by driving experience (years) and the number of days a driver works in a year^28^.

7 $${\text{CNE}} = {\text{E}} \times \left( {{\text{Years}}\;{\text{of}}\;{\text{working}}} \right) \times \left( {{\text{Days}}/{\text{year}}} \right)$$

where E = Total daily noise exposure, in Pa^2^ S, Days/year = Total days in year − total non-driving days in a year, The unit of CNE is pascals squared seconds (Pa^2^ S), To make the data of CNE simple (to reduce the range), the CNE in pascals squared seconds (Pa^2^ S) was converted into Pascals squared hours i.e., CNE (Pa^2^ Hrs) unit:

8 $$CNE \left( {{\text{Pa}}^{2} \;{\text{Hrs}}} \right) = CNE \left( {{\text{Pa}}^{2} \;{\text{S}}} \right)/3600$$

In the next step, this CNE (Pa^2^ Hrs) was further converted into CNE (dB(A) Hrs) by the following equation:

9 $$CNE \left( {{\text{dB}} \left( {\text{A}} \right)\;{\text{Hrs}}} \right) = 10\log \times \frac{{CNE \left( {{\text{Pa}}^{2} \;{\text{Hrs}}} \right)}}{{p_{ref}^{2} }}$$

### Medical examination

#### Otoscopic examination

Before conducting a pure tone audiometric test, the otoscopic examination of both ears of each subject was done. This otoscopic examination is done to rule out any possibility of chronic suppurative otitis media (CSOM) or other medical problems of the outer and inner ear. The subjects who found fit in the otoscopic examination were assessed for hearing ability by performing pure tone audiometry.

#### Pure tone audiometry test

The pure tone audiometry of the ears of 300 subjects (150 tempo drivers as the exposed noise group and 150 car drivers as the unexposed noise group) was conducted a minimum of 12 h after the last noise exposure to exclude the effect of temporary threshold shift.

A medical Grade MAICO (Model: MA42) Audiometer was used for audiometric testing. The product specifications of the instrument were; Type of audiometer: Diagnostic; Usage: Hospital, Clinic; Grade: Medical Grade; Features: Portable; Brand: Maico; Model Number: MA42; Weight (kg): 0.285 (with battery); Voltage (V): 110–220 V; Screen Size: 2.5–4 Inch.

Testing was carried out by conducting air conduction tests at frequencies of 250, 500, 1000, 2000, 3000, 4000, 6000 and 8000 Hz, in 5 dB(A) increments/ decrements taken for each ear in a soundproof room. Testing was carried out by conducting air conduction tests at frequencies of 250, 500, 1000, 2000, 3000, 4000, 6000 Hz and 8000 Hz, in 5 dB(A) increments/decrements taken for each ear in a soundproof room. Audiometry was done using the method recommended by the American Speech and Hearing Association. In all over the world, different countries practice different criteria to define hearing loss. Among them three major criteria i.e., World Health Organization (WHO criteria), National Institute for Occupational Safety and Health (NIOSH criteria) and Occupational Safety and Health Administration (OSHA criteria) loss are mostly used worldwide^29^. In present study above three criteria have been explored to define hearing loss. WHO criteria consider the arithmetic average of better ear audiometric hearing threshold shifts at frequencies 500, 1000, 2000 and 4000 Hz. It is also being followed in India. In contrast, hearing loss assessment in the NIOSH criteria takes into account the arithmetic average of both ears' audiometric hearing threshold shifts at frequencies of 1000, 2000, 3000 and 4000 Hz. Similar to this, OSHA guidelines take into account, the arithmetic average of both ears' audiometric hearing threshold shifts at frequencies of 1000, 2000 and 3000 Hz.

### Questioners

Pre-tested interview schedule was used to collect the information of socio-demographic characteristics, substance abuse, time-activity pattern, self-reported hearing problems, general information, physical health outcome at the end of working hours and any suggestion/query from the drivers.

### Ethical approval

The study was approved by the Institutional Ethics Committee of King George’s Medical University Lucknow, vide letter No. 2061/Ethics/R.cell-17 Dated 20/11/17. The registration no. of the ethics committee is ECR/262/Inst/UP/2013/RR-16 and reference code for the said approval is 86th ECM II B-Ph.D./P3. All methods were performed in accordance with the relevant guidelines and regulations. Informed consent was obtained from all subjects and their legal guardian(s).

## Results and discussions

### Noise quality data

Out of the three selected routes, on route no. 1 both diesel and CNG tempos are plying however, at route no. 2 only CNG and at route no. 3 only diesel tempo is plying. In-vehicle time-weighted average (TWA) equivalent noise level has been calculated for diesel and CNG tempos on all routes and no significant difference has been noticed. In-vehicle TWA equivalent noise levels (L_Aeq_) for diesel tempos are 86.26 dB(A) [L_10_ 89.6, L_50_ 83.1 and L_90_ 76.5 dB(A)] and for CNG tempos are 83.99 dB(A) [L_10_ 86.6, L_50_ 81.5 and L_90_ 75.5 dB(A)]. Similarly, in the waiting area, the TWA equivalent noise level at all routes have been calculated and no such difference has been observed among the diesel tempo stand, CNG tempo stand and combined tempo stand (Diesel and CNG), their values have been observed as 84.13 dB(A) [L_10_ 87.1, L_50_ 79.9 and L_90_ 73.4 dB(A)], 83.78 dB(A) [L_10_ 86.0, L_50_ 78.3 and L_90_ 71.1 dB(A)] and 84.32 dB(A) [L_10_ 87.6, L_50_ 79.8 and L_90_ 72.6 dB(A)] respectively.

However, a significant difference has been observed in the unexposed group i.e., private car drivers. In-vehicle TWA equivalent noise level in cars has been measured as 74.63 dB(A) [L_10_ 77.0, L_50_ 69.9 and L_90_ 62.3 dB(A)], which is quite less than the tempos.

### Cumulative noise exposure

CNE has been calculated for both exposed and unexposed groups. To understand the association of CNE with age groups, an unpaired t-test has been performed over tempo and car drivers. CNE was found to be significantly different (*p* < 0.001) in all age groups of exposed and unexposed (Table 1).

**Table 1:** Association of CNE (Pa^2^ Hrs) among tempo and car drivers by their age groups (in years).

Age (years)	CNE tempo driver		CNE car drivers		t value	*p* value^a^
	Mean ± SD	Range	Mean ± SD	Range		
< 26	2507.97 ± 2009.94	302.32–6946.10	138.23 ± 73.33	33.92–262.07	6.66	< 0.001
26–30	2206.39 ± 1184.89	785.05–5483.48	219.98 ± 89.01	44.25–398.21	6.69	< 0.001
31–35	4099.76 ± 1882.19	835.62–7352.51	421.59 ± 130.06	175.40–642.32	10.32	< 0.001
36–40	4491.02 ± 2663.93	604.44–10,893.17	398.42 ± 207.09	112.32–830.78	7.65	< 0.001
41–45	6224.60 ± 3694.68	650.50–17,154.10	576.92 ± 232.41	300.04–1113.40	7.78	< 0.001
46–50	6492.87 ± 3893.07	1781.79–15,516.72	648.95 ± 216.86	214.03–1004.05	7.18	< 0.001

^a^Unpaired t-test.

### Audiometry test

Audiometry test has been performed for both ears and both exposed and unexposed group. To understand the association of hearing loss frequencies, an unpaired t-test has been performed over tempo and car drivers. A statistically significant difference in the hearing threshold levels in the both ears of tempo drivers and car drivers was found at frequencies of “250, 500, 1000, 2000, 3000, 4000, 6000 and 8000 Hz” (Table 2). The group mean hearing loss in tempo drivers and car drivers was found to be the maximum at a frequency of 4000 Hz (4 kHz.), which could be a sign of noise-induced hearing loss, as hearing loss first occurs at 4000 Hz due to noise exposure.

**Table 2:** Hearing loss (dB) by Audiometry at different frequencies.

Freq (KHz)	Right ear			Left ear		
	Tempo drivers (Mean ± SD)	Car drivers (Mean ± SD)	*p* value^a^	Tempo drivers (Mean ± SD)	Car drivers (Mean ± SD)	*p* value^a^
0.25	16.23 ± 11.94	7.76 ± 8.68	< 0.01	17.83 ± 13.75	11.86 ± 10.40	< 0.01
0.50	20.70 ± 12.19	12.73 ± 9.72	< 0.01	15.33 ± 12.44	11.30 ± 8.74	< 0.01
1	18.20 ± 12.45	10.63 ± 6.93	< 0.01	18.40 ± 12.78	13.20 ± 8.36	< 0.01
2	25.73 ± 12.24	14.45 ± 6.43	< 0.01	23.53 ± 11.24	17.17 ± 6.96	< 0.01
3	31.07 ± 14.78	16.40 ± 7.39	< 0.01	30.20 ± 14.35	18.90 ± 8.17	< 0.01
4	34.97 ± 15.23	18.40 ± 7.63	< 0.01	34.87 ± 15.34	22.03 ± 8.59	< 0.01
6	30.37 ± 17.38	14.80 ± 8.78	< 0.01	30.23 ± 17.87	16.83 ± 10.14	< 0.01
8	30.87 ± 19.97	13.67 ± 10.67	< 0.01	32.50 ± 22.51	16.63 ± 13.54	< 0.01

^a^Unpaired t-test.

### Hearing loss

To understand the association between age and hearing loss, an unpaired t-test has been performed over tempo and car drivers as per WHO criteria for hearing loss. Calculation of hearing loss (adjusted for HL in car drivers) in individual (tempo driver): A tempo driver of age 46 years had hearing loss (NIOSH criteria) of 26.25 dB, for the corresponding age (46 years) of car driver had hearing loss (NIOSH criteria) of 19.50 dB (calculated). Then hearing loss, HL (adjusted for HL in car drivers) of Tempo drivers was calculated by deducting HL of car driver from HL of tempo driver i.e., 26.25 − 19.50 dB = 6.75 dB. Therefore, HL (adjusted for HL in car drivers) of Tempo drivers = 6.75 dB.

It has been analyzed that the average hearing threshold shift in age group upto 30 years in tempo drivers was 15.44 ± 5.45 dB and in car drivers, it was 11.27 ± 4.07 dB. Drivers of the age group 31 to 40 years showed an average threshold shift of 19.74 ± 5.83 and 12.55 ± 3.75 dB among tempo and car drivers, respectively. Also, the drivers in the age group of 41 to 50 years showed mean hearing threshold shift of 28.26 ± 8.96 and 16.17 ± 7.73 dB among tempo and car drivers, respectively (Table 3). The difference in tempo and car drivers hearing threshold shift was statistically significant across all three age groups (Table 4).

**Table 3:** Association of average hearing threshold shift* (dB) in better ear (WHO criteria of HL).

Age groups in completed years (Years)	The average threshold shifts of hearing (dB) in better ear*		t value	*p* value^a^
	Tempo Drivers (Mean ± SD)	Car Drivers (Mean ± SD)		
≤ 30	15.44 ± 5.45	11.27 ± 4.07	4.18	< 0.01
31–40	19.74 ± 5.83	12.55 ± 3.75	7.64	< 0.01
41–50	28.26 ± 8.96	16.17 ± 7.73	7.14	< 0.01
Overall (in all age groups)	21.15 ± 8.65	13.34 ± 5.79	9.17	< 0.01

*At combined frequencies of 500, 1000, 2000 and 4000 Hz.

^a^Unpaired t-test.

**Table 4:** Regression analysis models using CNE to estimate hearing loss (HL) among tempo drivers using WHO criteria (Model 1).

Variables (n = 150)	Unstandardized coefficient		Standardized coefficient	*p* value	Confidence interval (95%)	
	B	S.E	Β		Lower	Upper
Model 1 HL (WHO criteria), Average hearing threshold shift at frequencies of 0.5, 1, 2, 4 kHz (better ear), r^2^ = 0.551 F = 92.55, *p* < 0.001						
Constant	4.094	1.898		0.033	0.344	7.845
Age	0.309	0.060	0.330	< 0.001	0.189	0.429
CNE (P^2^ Hrs)	0.001	0.000	0.517	< 0.001	0.001	0.002

To further investigate the hearing loss in the exposed group (tempo drivers), a regression analysis model has been done using CNE to estimate hearing loss among tempo drivers. This analysis was done keeping hearing loss among tempo drivers as the dependent variable and age (years), Energy (in Pa^2^ Hrs) as independent variables using three different criteria of hearing loss definitions, i.e., WHO criteria, NIOSH criteria and OSHA criteria. It was observed that in all the models, both the independent variables were highly statistically significant in predicting hearing loss. Also, all the models could explain the total variation in dependent variable by independent variable quite well, i.e., 55.1, 68.1, and 63.37% by WHO Criteria, NIOSH Criteria and OSHA criteria respectively. Furthermore, the independent variables best explained a NIOSH (Model 2: Table 5 criterion of hearing loss as compared to WHO criteria and OSHA criteria of hearing loss (Tables 4 and 6).

**Table 5:** Regression analysis models using CNE to estimate hearing loss (HL) among tempo drivers using NIOSH criteria (Model 2).

Variables (n = 150)	Unstandardized coefficient		Standardized coefficient	*p* value	Confidence interval (95%)	
	B	S.E	Β		Lower	Upper
Model-2: HL (NIOSH criteria), Average hearing threshold shift at frequencies of 1, 2, 3, 4 kHz (both ears), r^2^ = 0.681F = 164.85, *p* < 0.001						
Constant	5.942	1.883		0.002	2.221	9.662
Age	0.351	0.060	0.316	< 0.001	0.232	0.470
CNE (P^2^ Hrs)	0.002	0.000	0.620	< 0.001	0.002	0.002

**Table 6:** Regression analysis models using CNE to estimate hearing loss (HL) among tempo drivers using OSHA criteria (Model 3).

Variables (n = 150)	Unstandardized coefficient		Standardized coefficient	*p* value	Confidence interval (95%)	
	B	S.E	Β		Lower	Upper
Model-3: HL (OSHA criteria), Average hearing threshold shift at frequencies of 1, 2, 3 kHz (both ears), r^2^ = 0.633 F = 126.56, *p* < 0.001						
Constant	5.251	2.035		0.011	1.229	9.274
Age	0.301	0.065	0.273	< 0.001	0.172	0.429
CNE (P^2^ Hrs)	0.002	0.000	0.616	< 0.001	0.002	0.002

Therefore, for further analysis only NIOSH criteria of hearing loss was adopted (Table 7).

**Table 7:** Predictive ability of the model-2 (NIOSH criteria) for hearing loss (dB) as dependent, CNE (Pa^2^ Hrs) and age (years) as independent.

Probability cutoff	Hearing loss (dB) (NIOSH criteria)				Total	
	Yes		No			
	No	%	No	%	No	%
> 25%	65	43.3	19	12.7	84	56
≤ 25%	11	7.3	55	36.7	66	44
Total	76	50.7	74	49.3	150	100
Predictive values, % (95%CI)						
Sensitivity	85.5 (77.6–93.4)					
Sensitivity	74.3 (64.4–84.3)					
PPV	77.4 (68.4–86.3)					
NPV	83.3 (74.3–92.3)					
AUC (95%CI)	0.90 (0.85–0.94)					

The model cutoff value > 25% correctly predicted hearing loss in 43.3% subjects with sensitivity and specificity of 85.5% and 74.3% respectively. The area under the curve (AUC) for hearing loss was 0.90 (95% CI = 0.85–0.94). Multiple regression analysis was done to understand the dose (cumulative noise exposure)-response (hearing loss) relationship in exposed group (Tables 4, 5, 6), considering hearing loss as dependent variable, age and cumulative noise exposure as independent variable which showed a positive association of hearing loss with age and cumulative noise exposure.

**Figure 2:**
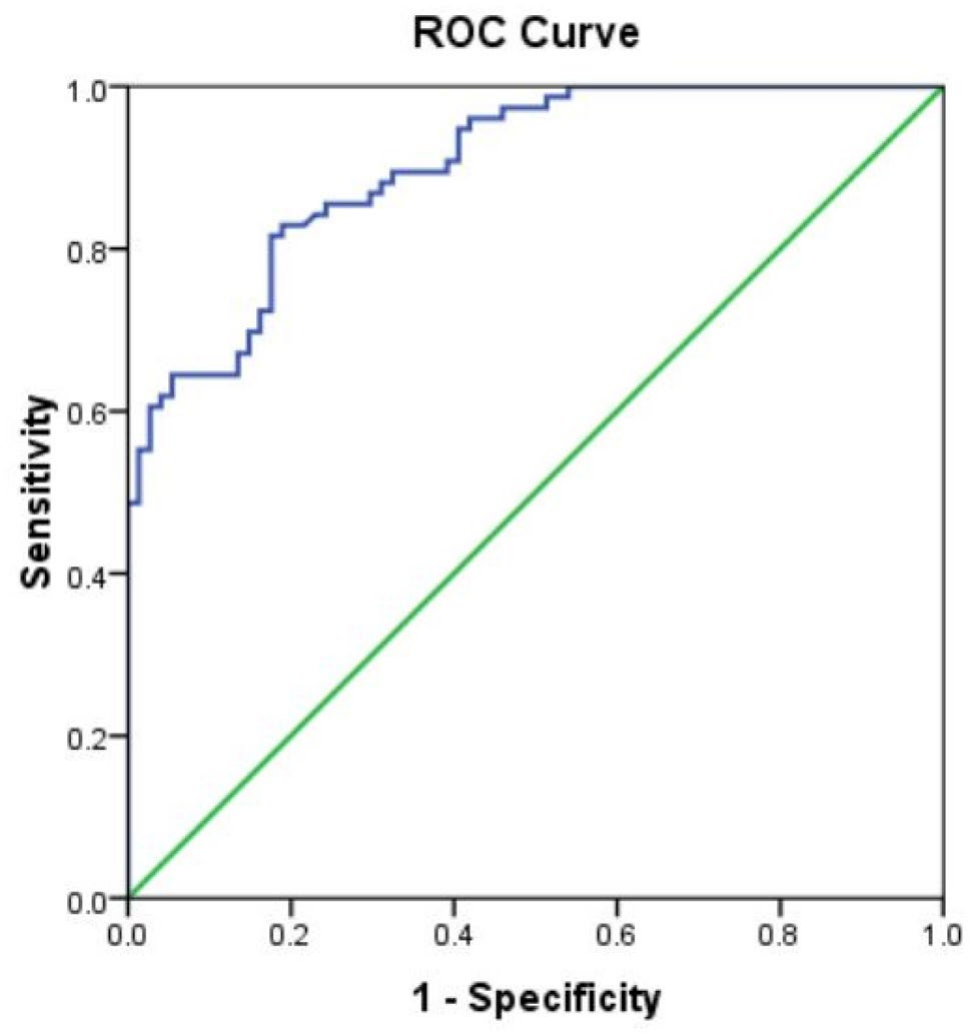
ROC curve showing predictive values for haring loss (NIOSH) for CNE (Pa^2^ Hrs) and age (Yrs.) of tempo drivers.

In this study three different criteria of hearing loss definitions i.e., WHO criteria, NIOSH criteria and OSHA criteria were used. It was found that in all the models both the independent variables were statistically significant in predicting the hearing loss. Also, all the models could explain total variation in dependent variable by independent variable quite well i.e., R^2^ = 0.633, 0.681 and 0.541 by WHO Criteria, NIOSH Criteria and OSHA criteria respectively. Similar study was done by Xie et al.^11^ and reported R^2^ = 0.386 i.e., their model could explain hearing loss by age and CNE 38.6%. However, in this study the best model (model 2) could explain total variation in dependent variable by independent variables quite well i.e., 68.1%. The difference in R^2^ values could be because of different definition of hearing loss, they took average of high frequency pure tone audiometric threshold shift at 3000, 4000 and 6000 frequencies; however, in this study NIOSH criteria of hearing loss was used. As NIOSH (Model 2) criterion of hearing loss was found best thus for further analysis only Model 2 has been taken as final model. This model clearly indicates that hearing loss occurs due to noise exposure along with the age of the subjects i.e., aging. To get the relationship between hearing loss and CNE, the hearing loss due to ageing must be logically removed. Non-commercial car drivers were taken as unexposed group for this purpose. But when hearing loss in car drivers were plotted with their age, the data was found scattered (Fig. 2), R^2^ = 0.1785 which shows poor association between hearing loss and age of the car drivers, which shows some other variables are responsible for hearing loss in car drivers. The reason for poor association indicates that either drivers were exposed to some non-occupational noise or other unknown reasons. During planning phase of the study, so many discussions with experts were made, on selection of unexposed group and so many options were discussed, finally it was unanimously decided to go for car drivers. However, our study proceeded for further analysis with cars drivers hearing data and additionally, data given by Dobie et al.^30^ for age corrections at different frequencies due to aging. Two separate multiple regression analysis (i) hearing loss (adjusted with hearing loss in car drivers) as dependent variable and CNE as independent variable and (ii) hearing loss {(adjusted with hearing loss given by Dobie et al.^30^} as dependent variable and CNE (P^2^ Hrs) as independent variable were done. For both the conditions (i) and (ii), results showed a positive association with the cumulative noise exposure (P^2^ Hrs) with R^2^ = 0.571 and 0.564 respectively. Both the models explained that there is a relationship between hearing loss and cumulative noise exposure of the drivers (exposed group).

### Dose response between CNE and HL

**Figure 3:**
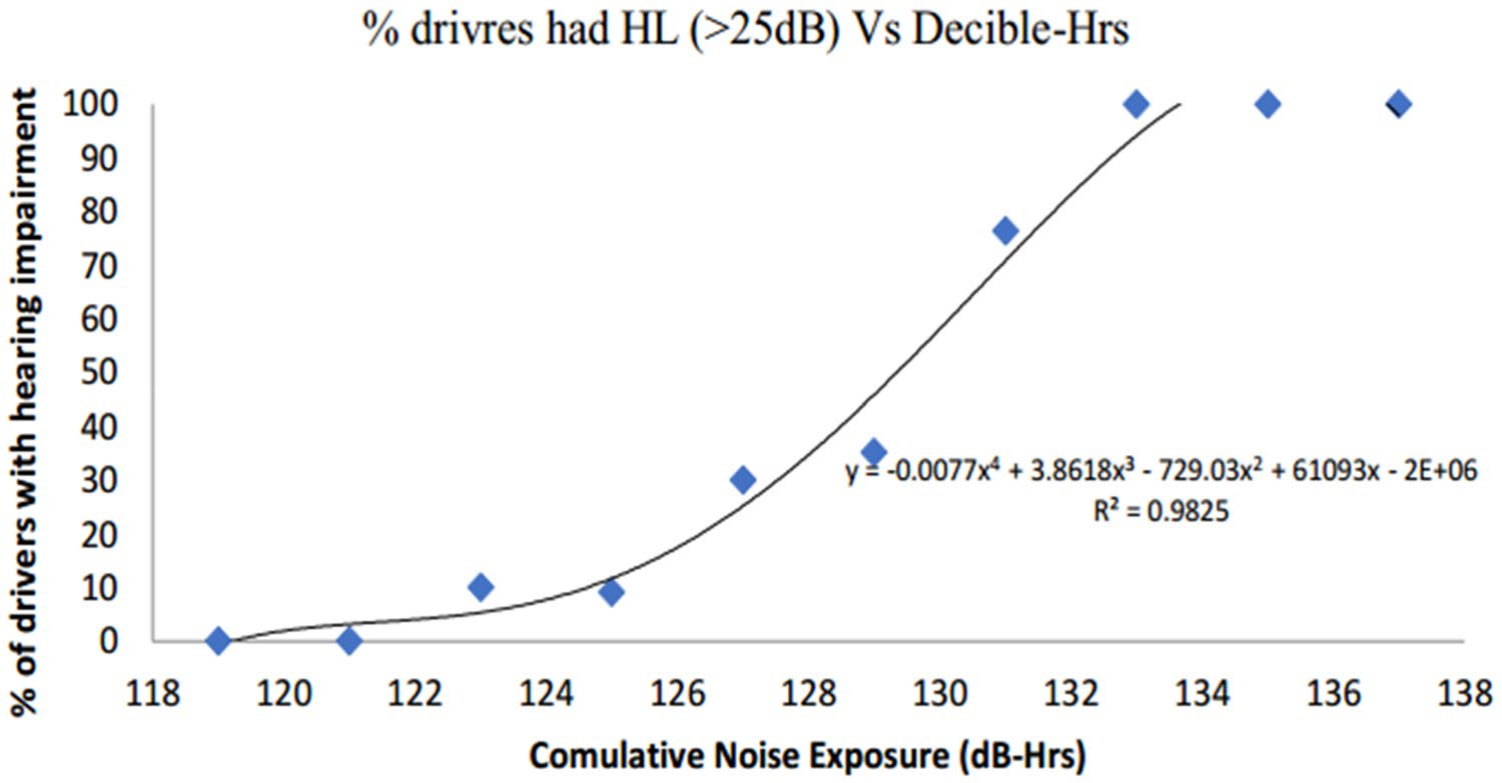
The dose response relationship between CNE (dB(A) Hrs) and percentage of driver with hearing impairment.

A curve has been plotted between Hearing loss and CNE (Pa^2^ Hr) and a linear relationship is observed between them (Fig. 3). As the range of cumulative noise exposure in pascals squared hour (Pa^2^ Hr) is too vast {302.32 (Pa^2^ Hrs) to 17,154.11 (Pa^2^ Hrs)} to distribute this CNE (Pa^2^ Hr) into groups according to hearing impairment (> 25 dB hearing loss) of tempo drivers^31^. The unit of CNE is converted from pascals square hour (Pa^2^ Hr) to (dB(A) Hr) and further, CNE (dB(A) Hr) were grouped by 3 dB(A) intervals (Table 8).

**Table 8:** Cumulative noise exposure (dB(A) Hr) and hearing impairment (HL > 25 dB) among tempo drivers.

CNE (dB(A) Hr)	No. of drivers	Tempo drivers with hearing impairment (HL > 25 dB)		Mean decibel
		Number	%HL > 25	
118–120	2	0	0	119
120–122	5	0	0	121
122–124	10	1	10	123
124–126	11	1	9.09	125
126–128	20	6	30	127
128–130	37	13	35.13	129
130–132	38	29	76.32	131
132–134	17	17	100	133
134–136	9	9	100	135
136–138	1	1	100	137

From the data of 150 drivers of the exposed group, number of drivers corresponding to 3 dB(A) cumulative noise exposure (dB(A) Hrs) interval had hearing impairment (HL > 25 dB) was retrieved and the percent of drivers with hearing impairment was calculated for each group^32^.

It can be observed that CNE (dB(A) Hrs) up to 121 dB(A) (approximate), the percentage of drivers with hearing impairment is zero, thereafter percentage increases with increase in CNE (dB(A) Hrs) and hearing loss reach to 100% at CNE (dB(A) Hrs) of 133 dB(A) and thereafter it remained 100% with respect to increase in (dB(A) Hrs) (Fig. 3). By this analysis we can conclude that 121 dB(A) cumulative noise exposure (dB(A) Hrs) is 100% safe for one hour during lifetime and 133 dB(A) cumulative noise exposure (dB(A) Hrs) is 100% unsafe for one hour during lifetime (Table 8).

The relationship of percentage populations having hearing impairment (hearing loss > 25 dB) with the commutative noise exposure. CNE (dB(A) Hr) were grouped by 3 dB(A) intervals (Table 8) and dose–response curve between CNE (dB(A) Hrs) and percentage of driver with hearing impairment was generated (Fig. 3). Xie et al.^11^ have used the kurtosis-adjusted cumulative noise exposure metric in evaluating the hearing loss risk for complex noise and found the similar trends. Zhang et al.^13^ have found the similar curve trends as presented in our study.

As per NIOSH criteria, 3-dB(A) exchange rate for time-intensity trade off was used as it explains that the energy of noise gets doubled, when there is an increase in noise level by 3 dB(A), likewise energy of noise gets halved, when there is a decrease in noise level by 3 dB(A). Based on the above discussion two conclusions can be made from the results^33^.

I. 121 dB(A) noise exposure is 100% safe for one hour during lifetime and
II. 133 dB(A) noise exposure is 100% unsafe for one hour during lifetime.

As 121 dB(A) noise level is safe for one hour, the 118 dB(A) noise level will be safe for 2 h, 115 dB(A) noise level will be safe for 4 h, 112 dB(A) noise level will be safe for 8 h and so on. Likewise, as the 133 dB(A) noise levels is unsafe for one hour, the 130 dB(A) noise will be unsafe for 2 h, 127 dB(A) noise will be unsafe for 4 h, 124 dB(A) noise will be unsafe for 8 h and so on^34^. Based on the above data of safe and unsafe lifetime exposure limits for different durations were regressed and two models were derived:

1. Unsafe Noise Level (dB(A)) = − 4.335 ln (lifetime noise exposure duration in hours) + 133.02, R^2^ = 1
2. Safe Noise Level (dB(A)) = − 4.335 ln (lifetime noise exposure duration in hours) + 121.02, R^2^ = 1

From above two equations, safe or unsafe noise exposure levels for different durations or vice versa can be calculated. It may be useful for different statutory bodies and policy makers to form rule to abate noise pollution at work place.

### Strengths and limitations

#### Strengths

A pre-tested interview schedule was used to collect in depth information on socio-demographic characteristics, substance abuse, Time-Activity patterns, Self-reported hearing problems, General information, Physical health outcome at the end of working hours and any suggestion/ query from the drivers. Strict inclusion and exclusion criteria were followed, and otoscopic examinations of every driver have been done by experienced otorhinolaryngologists. The present study derived two model equations to predict safe and unsafe levels of noise with time exposure.

#### Limitations

In this study only, occupational noise was considered, but the subjects (drivers) may be exposed to several non-occupational noises in their lifetime, which was not included in the study as it was practically not feasible. During the in-vehicle noise monitoring period, drivers were requested not to play the music system. In this way, the study underestimated the cumulative noise exposure for those fond of music. The car drivers considered an unexposed group in our study, may be exposed to social or other noises. Hence, car drivers are not ideally an unexposed population.

## Conclusions

In the present research, it has been noticed that in vehicle time weighted average equivalent noise level is observed to equal in both diesel and CNG based tempos i.e., 86.26 and 83.99 dB(A); similar observation has also been noticed in tempo stand waiting areas for all tempo stands i.e., diesel tempo stand, CNG tempo stand and combined tempo stand (Diesel and CNG), their values have been observed as 84.13, 83.78 and 84.32 dB(A), respectively. However, in private cars, the in-vehicle time weighted average equivalent noise level is significantly lower compared to tempos, i.e., 74.63 dB(A). The research also investigated the cumulative noise exposure concerning age for exposed and unexposed groups and observed significant differences (*p* < 0.001) in all exposed and unexposed age groups. For audiometric loss among three different models, NIOSH model has been found best with R^2^ 68.1%. A dose response curve between hearing loss and Cumulative Noise Exposure has also been drawn and it has been investigated that till 123 dB(A), zero percent of drivers were found suffering from hearing loss, whereas, after 132 dB(A), 100% drivers have been found suffering from hearing loss. Based on this, safe and unsafe noise levels concerning exposure in hours have been derived, which helps policy makers decrease noise pollution and its exposure to individuals in the workplace.

## Data Availability

The datasets used and/or analyzed during the current study are available from the corresponding author on reasonable request.

## References

[1] Mondal NK, Dey M, Datta JK (2014). Vulnerability of bus and truck drivers affected from vehicle engine noise. Int. J. Sustain. Built Environ..

[2] Ang LYL, Cui F (2022). Remote work: Aircraft noise implications, prediction, and management in the built environment. Appl. Acoust..

[3] Loukzadeh Z, Shahrad S, Shojaoddiny-Ardekani A, Mehrparvar AH, Alamdarian M (2019). Effect of combined exposure to noise and vibration on hearing. Indian J. Occup. Environ. Med..

[4] Charrier I, Matheron N, Jouventin P (2001). Newborns need to learn their mother’s call before she can take off on a fishing trip. Nature.

[5] Shukla SP, Sageer S, Singh D, Markandeya (2023). A GIS based vehicular emission inventory including fugitive dust emissions of Lucknow city, India. Environ. Dev. Sustain..

[6] Markandeya, Verma PK, Mishra V, Singh NK, Shukla SP, Mohan D (2021). Spatio-temporal assessment of ambient air quality, their health effects and improvement during COVID-19 lockdown in one of the most polluted city of India. Environ. Sci. Pollut. Res..

[7] Sakhvidi MJZ, Sakhvidi FZ, Mehrparvar AH, Foraster M, Dadvand P (2018). Association between noise exposure and diabetes: A systematic review and meta-analysis. Environ. Res..

[8] Radun J, Maula H, Saarinen P, Keränen J, Alakoivu R, Hongisto V (2022). Health effects of wind turbine noise and road traffic noise on people living near wind turbines. Renew. Sustain. Energy Rev..

[9] Singh NK, Markandeya, Manar MK, Shukla SP, Mohan D (2023). Statistical and spatio-temporal analysis of noise pollution level & its health impact. Environ. Sci. Pollut. Res..

[10] Nandi SS, Dhatrak SV (2008). Occupational noise-induced hearing loss in India. Indian J. Occup. Environ. Med..

[11] Xie HW, Qiu W, Heyer NJ, Zhang MB, Zhang P, Zhao YM, Hamernik RP (2016). The use of the kurtosis-adjusted cumulative noise exposure metric in evaluating the hearing loss risk for complex noise. Ear Hear..

[12] Carter L, Black D, Bundy A, Williams W (2016). An estimation of the whole-of-life noise exposure of adolescent and young adult Australians with hearing impairment. J. Am. Acad. Audiol..

[13] Zhang H, Li N, Yang QL, Qiu W, Zhu LL, Tao LY, Davis RI, Heyer N, Zhao YM (2015). Comparison of two dose-response relationship of noise exposure evaluation results with high frequency hearing loss. Chin. Med. J..

[14] Sharif A, Taous A, Siddique BH, Dutta PG (2009). Prevalence of noise induced hearing loss among traffic police in Dhaka Metropolitan City. Mymensingh Med. J..

[15] Ansari H, Ansari-Moghaddam A, Mohammadi M, Tabatabaei SM, Fazli B, Pishevare-Mofrad M (2016). Status of hearing loss and its related factors among drivers in Zahedan, South-Eastern Iran. Glob. J. Health Sci..

[16] Beheshti MH, Firoozi CA, Alinaghi LAA (2016). Assessment of hearing loss among taxi and agency drivers in a city in northern Iran in 2011. J. Occup. Health Epidemiol..

[17] Voss S, Schneider A, Huth C, Wolf K, Markevych I, Schwettmann L, Rathmann W, Peters A, Breitner S (2021). Long-term exposure to air pollution, road traffic noise, residential greenness, and prevalent and incident metabolic syndrome: Results from the population-based KORA F4/FF4 cohort in Augsburg, Germany. Environ. Int..

[18] Turcot A, Girard SA, Courteau M, Baril J, Larocque R (2015). Noise-induced hearing loss and combined noise and vibration exposure. Occup. Med..

[19] Sørensen M, Poulsen AH, Hvidtfeldt UA, Brandt J, Frohn LM, Ketzel M, Christensen JH, Im U, Khan J, Münzel T, Raaschou-Nielsen O (2022). Air pollution, road traffic noise and lack of greenness and risk of type 2 diabetes: A multi-exposure prospective study covering Denmark. Environ. Int..

[20] Shin S, Bai LI, Oiamo TH, Burnett RT, Weichenthal S, Jerrett M, Kwong JC, Goldberg MS, Copes R, Kopp A, Chen H (2020). Association between road traffic noise and incidence of diabetes mellitus and hypertension in Toronto, Canada: A Population-Based Cohort Study. J. Am. Heart Assoc..

[21] Bai LI, Chen H, Hatzopoulou M, Jerrett M, Kwong JC, Burnett RT, van Donkelaar A, Copes R, Martin RV, Van Ryswyk K, Lu H, Kopp A, Weichenthal S (2018). Exposure to ambient ultrafine particles and nitrogen dioxide and incident hypertension and diabetes. Epidemiology.

[22] Chen XC, Chuang HC, Ward TJ, Sarkar C, Webster C, Cao J, Hsiao TC, Ho KF (2021). Toxicological effects of personal exposure to fine particles in adult residents of Hong Kong. Environ. Pollut..

[23] Ramazzini B (2001). De morbis artificum diatriba [diseases of workers]. Am. J. Public Health.

[24] Anyogita S, Prakash A, Jain VK (2004). A study of noise in CNG driven modes of transport in Delhi. Appl. Acoust..

[25] Lopes AC, Otowiz VG, Barros De, Lopes PM, Lauris JRP, Santos CC (2012). Prevalence of noise-induced hearing loss in drivers. Int. Arch. Otorhinolaryngol..

[26] Kumar K, Jain VK (1994). A study of noise in various modes of transport in Delhi. Appl. Acoust..

[27] ANSI. Determination of Occupational Noise Exposure and Estimation of Noise-Induced Hearing Impairment (with Erratum). Available from: https://webstore.ansi.org/standards/asa/ansis34419962006 (1996).

[28] Campbell, N. A. & Reece, J. B. Biology. Benjamin Cummings. Available from: https://books.google.co.in/books/about/Biology.html?id=9pqXQgAACAAJ&redir_esc=y (2002).

[29] Occupational Noise Exposure-1910.95|Occupational Safety and Health Administration. Available from: https://www.osha.gov/pls/oshaweb/owadisp.show_document?p_id=9735&p_table=standards.

[30] Dobie RA, Wojcik NC (2015). Age correction in monitoring audiometry: Method to update OSHA age-correction tables to include older workers. BMJ Open.

[31] Chepesiuk R (2005). Decibel hell: The effects of living in a noisy world. Environ. Health Perspect..

[32] Chen Y, Zhang M, Qiu W, Sun X, Wang X, Dong Y, Chen Z, Hu W (2019). Prevalence and determinants of noise-induced hearing loss among workers in the automotive industry in China: A pilot study. J. Occup. Health.

[33] Beutel ME, Jünger C, Klein EM, Wild P, Lackner K, Blettner M, Binder H, Michal M, Wiltink J, Brähler E, Münzel T (2016). Noise annoyance is associated with depression and anxiety in the general population the contribution of aircraft noise. PLoS One.

[34] Banerjee D (2013). Road traffic noise exposure and annoyance: A cross-sectional study among adult Indian population. Noise Health.

